# Supplementary material for: Evaluating the Impact of Outcome Delay on the Efficiency of Sample Size Re‐Estimation
Source: Pharm Stat. 2026 Sep 24;25(6):e70117. doi: 10.1002/pst.70117 (PMC13602115; doi:10.1002/pst.70117)
Supplement: Supplementary file 1 [file PST-25-0-s001.pdf]

Supplementary materials for "Evaluating the impact of outcome delay on the efficiency of sample size re-estimation"

1 Impact of delay on SSR design with continuous treatment outcomes

This section contains the exact values for *delay impact*, $RMSE$, the cost metric and empirical power values for different m values (in particular, $m = 0, 3, 6, 9, \ldots, 24$). Table 1 provides these values along with the oracle sample size and average final sample sizes from the 10,000 simulation under a uniform recruitment assumption, whereas, tables 2 and 3 provide the same for a mixed and linearly increasing recruitment pattern.

It can be seen from tables 1-3 that, the average re-estimated sample size increases with delay. This is because, as the *delay impact* increases a greater proportion of trials ends up with $n_1 + n_{delay}$ sample. This gives a rise to the minimum attainable re-estimated sample size thus increasing the average re-estimated sample size.

For trials in which $\sigma^2_{init} > \sigma^2$, they tend to become overpowered quickly due to the increase in the sample size. The power can increase to as big as 94% from 80% for a sufficiently large delay (24 months). The power increases from 80 to 89% for a trial with correctly specified $\sigma^2_{init} = \sigma^2$ over increasing delay lengths. The power remains relatively constant for the third scenario. However, for linear recruitment, the power is further influenced due to the increase in pipeline subjects.

Table 1: Impact of delay (m) on Efficiency and cost parameters for a SSR design with Uniform recruitment. Here, all the 10,000 simulated designs for every m maintain $\alpha = 0.05, \beta = 0.2, \delta_0 = \delta = 0.35$. The initial sample size is computed based on $\sigma_0^2 = 1$ as 202 and the interim is conducted after 70 patients are recruited across both arms.

Case	Empirical Power	m	n_{oracle}	Average N^*	n_{delay}	Average σ^*2	$RMSE_{single}$	$RMSE_{SSR}$	$Cost_{single}$	$Cost_{SSR}$	Delay Impact
I	0.80	0	163.52	170.8	0	0.91	38.36	29.88	17.17	10.71	0.000
	0.80	3		170.88	25	0.91		29.70		10.58	0.001
	0.80	6		171.27	50	0.91		29.25		10.21	0.022
	0.80	9		173.18	76	0.91		26.60		8.28	0.190
	0.83	12		182.3	101	0.91		25.94		7.80	0.532
	0.87	15		199.45	126	0.91		37.28		16.03	0.805
	0.89	18		221.71	151	0.91		58.34		38.10	0.952
	0.92	21		247.1	177	0.91		83.59		75.99	0.991
	0.94	24		272.01	202	0.91		108.49		125.25	0.999
II	0.80	0	201.88	209.07	0	1.01	0	35.95	0.00	15.63	0.000
	0.80	3		208.88	25	1.01		36.06		15.73	0.000
	0.81	6		209.21	50	1.01		36.42		16.02	0.001
	0.80	9		209.83	76	1.01		35.63		15.25	0.024
	0.81	12		210.97	101	1.01		33.75		13.44	0.142
	0.82	15		217.63	126	1.01		30.74		10.91	0.360
	0.84	18		230.63	151	1.01		34.33		13.66	0.650
	0.86	21		250.15	177	1.01		49.45		28.07	0.857
	0.89	24		272.74	202	1.01		71.02		56.49	0.955
III	0.80	0	244.27	251.35	0	1.11	42.39	43.19	24.90	22.79	0.000
	0.80	3		251.42	25	1.11		43.19		22.73	0.000
	0.80	6		251.36	50	1.11		43.23		22.74	0.000
	0.79	9		251.75	76	1.11		43.29		22.81	0.002

Table 1: Impact of delay (m) on Efficiency and cost parameters for a SSR design with Uniform recruitment. Here, all the 10,000 simulated designs for every m maintain $\alpha = 0.05, \beta = 0.2, \delta_0 = \delta = 0.35$. The initial sample size is computed based on $\sigma_0^2 = 1$ as 202 and the interim is conducted after 70 patients are recruited across both arms.

Case	Empirical Power	m	n_{oracle}	Average N^*	n_{delay}	Average σ^*2	$RMSE_{single}$	$RMSE_{SSR}$	$Cost_{single}$	$Cost_{SSR}$	Delay Impact
	0.79	12		251.95	101	1.11		42.76		22.16	0.018
	0.81	15		252.92	126	1.11		41.25		20.32	0.085
	0.80	18		257.06	151	1.11		37.99		16.82	0.247
	0.82	21		265.93	177	1.11		35.18		14.27	0.483
	0.84	24		280.71	202	1.11		41.25		19.76	0.700

Table 2: Impact of delay (m) on Efficiency and cost parameters for a SSR design with Mixed recruitment. Here, all the 10,000 simulated designs for every m maintain $\alpha = 0.05, \beta = 0.2, \delta_0 = \delta = 0.35$. The initial sample size is computed based on $\sigma_0^2 = 1$ as 202 and the interim is conducted after 70 patients are recruited across both arms.

Case	Empirical Power	m	n_{oracle}	Average N^*	n_{delay}	Average σ^*2	$RMSE_{single}$	$RMSE_{SSR}$	$Cost_{single}$	$Cost_{SSR}$	Delay Impact
I	0.80	0	163.52	170.25	0	0.91	38.36	29.72	17.15	10.62	0.000
	0.80	3		171.31	28	0.92		29.83		10.65	0.001
	0.81	6		171.56	56	0.91		29.11		10.06	0.045
	0.81	9		175.84	85	0.91		26.04		7.87	0.307
	0.84	12		189.78	113	0.91		29.79		10.31	0.678
	0.88	15		212.46	141	0.91		49.35		27.61	0.913
	0.92	18		239.19	169	0.91		75.69		62.81	0.986
	0.94	21		267.01	197	0.91		103.49		114.40	0.999
	0.95	24		295.00	225	0.91		131.48		181.23	1.000
II	0.80	0	201.88	209.58	0	1.01	0.00	36.28	0.00	15.86	0.000
	0.80	3		209.13	28	1.01		35.88		15.56	0.000
	0.79	6		209.29	56	1.01		36.06		15.71	0.003
	0.80	9		209.96	85	1.01		35.13		14.76	0.049
	0.81	12		213.11	113	1.01		31.79		11.78	0.237
	0.83	15		224.14	141	1.01		30.93		11.06	0.545
	0.85	18		243.22	169	1.01		43.09		21.48	0.811
	0.88	21		268.14	197	1.01		66.53		49.87	0.936
	0.91	24		295.22	225	1.01		93.37		95.52	0.986
III	0.80	0	244.27	251.71	0	1.11	42.39	43.46	24.92	22.94	0.000
	0.80	3		251.98	28	1.11		43.31		22.83	0.000
	0.80	6		251.77	56	1.11		43.51		23.09	0.000
	0.80	9		251.06	85	1.11		43.03		22.54	0.005

Table 2: Impact of delay (m) on Efficiency and cost parameters for a SSR design with Mixed recruitment. Here, all the 10,000 simulated designs for every m maintain $\alpha = 0.05, \beta = 0.2, \delta_0 = \delta = 0.35$. The initial sample size is computed based on $\sigma_0^2 = 1$ as 202 and the interim is conducted after 70 patients are recruited across both arms.

Case	Empirical Power	m	n_{oracle}	Average N^*	n_{delay}	Average σ^*2	$RMSE_{single}$	$RMSE_{SSR}$	$Cost_{single}$	$Cost_{SSR}$	Delay Impact
	0.79	12		251.96	113	1.11		42.40		21.64	0.044
	0.80	15		254.38	141	1.11		39.18		18.09	0.175
	0.82	18		262.69	169	1.11		35.69		14.68	0.406
	0.84	21		277.53	197	1.11		39.41		18.00	0.661
	0.86	24		298.92	225	1.11		56.17		36.33	0.853

Table 3: Impact of delay (m) on Efficiency and cost parameters for a SSR design with Linear recruitment. Here, all the 10,000 simulated designs for every m maintain $\alpha = 0.05, \beta = 0.2, \delta_0 = \delta = 0.35$. The initial sample size is computed based on $\sigma_0^2 = 1$ as 202 and the interim is conducted after 70 patients are recruited across both arms.

Case	Empirical Power	m	n_{oracle}	Average N^*	n_{delay}	Average σ^*2	$RMSE_{single}$	$RMSE_{SSR}$	$Cost_{single}$	$Cost_{SSR}$	Delay Impact
I	0.80	0	163.52	170.65	0	0.91	38.36	29.61	17.16	10.54	0.000
	0.80	3		170.78	32	0.91		29.71		10.57	0.002
	0.80	6		172.19	70	0.91		27.78		9.06	0.131
	0.85	9		190.95	115	0.91		30.42		10.76	0.705
	0.91	12		233.35	163	0.91		69.90		53.91	0.975
	0.95	15		282.00	212	0.91		118.48		148.30	1.000
	0.97	18		330.00	260	0.91		166.48		285.87	1.000
	0.99	21		379.00	309	0.91		215.48		472.28	1.000
	0.99	24		427.00	357	0.91		263.48		700.67	1.000
II	0.80	0	201.88	209.60	0	1.01	0.00	36.17	0.00	15.77	0.000
	0.80	3		208.77	32	1.01		36.00		15.72	0.000
	0.80	6		210.07	70	1.01		35.93		15.54	0.014
	0.81	9		213.49	115	1.01		31.03		11.22	0.257
	0.85	12		238.85	163	1.01		39.69		18.26	0.760
	0.90	15		282.45	212	1.01		80.67		72.18	0.971
	0.93	18		330.01	260	1.01		128.14		175.29	0.998
	0.96	21		379.00	309	1.01		177.12		326.75	1.000
	0.97	24		427.00	357	1.01		225.12		519.72	1.000
III	0.80	0	244.27	251.27	0	1.11	42.39	43.49	24.89	23.08	0.000
	0.80	3		251.40	32	1.11		43.46		22.99	0.000
	0.80	6		251.78	70	1.11		42.90		22.41	0.001
	0.80	9		252.26	115	1.11		42.21		21.44	0.050

Table 3: Impact of delay (m) on Efficiency and cost parameters for a SSR design with Linear recruitment. Here, all the 10,000 simulated designs for every m maintain $\alpha = 0.05, \beta = 0.2, \delta_0 = \delta = 0.35$. The initial sample size is computed based on $\sigma_0^2 = 1$ as 202 and the interim is conducted after 70 patients are recruited across both arms.

Case	Empirical Power	m	n_{oracle}	Average N^*	n_{delay}	Average σ^*2	$RMSE_{single}$	$RMSE_{SSR}$	$Cost_{single}$	$Cost_{SSR}$	Delay Impact
	0.81	12		260.82	163	1.11		36.51		15.40	0.345
	0.85	15		288.43	212	1.11		47.24		25.86	0.767
	0.89	18		330.92	260	1.11		86.87		84.46	0.957
	0.93	21		379.06	309	1.11		134.80		196.25	0.997
	0.95	24		427.01	357	1.11		182.73		351.91	1.000

2 Impact of delay on SSR design with different treatment effects

In this section, we plot the distribution of the re-estimated sample sizes as well as the delay impact for different (initial) treatment effect assumptions. Figures 1 and 2 shows the re-estimated sample sizes under different recruitment models for a treatment effect of 0.2 and 0.5 respectively under the same σ^2 assumptions as in cases I, II and III. Figures 3 and 2 shows the re-estimated sample sizes under different recruitment models for a treatment effect of 0.2 and 0.5 respectively under the same σ^2 assumptions as in cases I, II and III. Here, therefore the true standardised effects are different from the main paper. Further, since under the treatment effect of 0.5, the total sample size required is 98 for both the arms. Hence doing a re-estimation after 70 first stage sample size is not practical. Therefore, the re-estimation is done after each arm has recruited 15 participants.

It can be observed from the figures that delay affects the distribution of the re-estimated sample sizes similarly for both the treatment effects. However, the delay impact starts to accumulate more quickly for a larger treatment effect. In this case, the required number of participants is relatively lower compared to the other two cases, therefore, the accumulated pipelines starts to quickly affect the re-estimated sample size often contributing to become the final sample size. Note that, the SSR is conducted in a blinded manner using one sample variance estimator, therefore, we assume the treatment effects are not re-estimated during the interim analysis. The underlying treatment effects are assumed to be the target treatment effects i.e. $\delta = \delta_0$.

We have also plotted the cost metric for understanding the efficiency lost due to delay. These results can be observed in figures 4 and 5. Here, one interesting finding is that, for a larger treatment effect, the cost almost always appears to be higher for a sample size re-estimation design compared to a single stage equivalent for all the cases. Although the magnitude of this difference is significantly low.

3 Impact of delay on SSR design with different inflection points for mixed recruitment pattern

In the main paper, we have discussed the impact of delay on SSR designs with mixed recruitment pattern. The underlying assumption in the recruitment pattern is. new centres open up facilitating a linearly increasing recruitment till 0.25 times of the total recruitment length, i.e. 6 months. In this section we provide further results for other recruitment scenario where the inflection point lies at 3, 6, 9 and 12 months through recruitment in a 24 month recruitment period. Figures 6 and 7 plots the distribution of reestimated sample size and delay impact for varying delay lengths for different values of the inflection point. It can be observed from the figures that longer the recruitment pattern remains linear, a higher number of pipelines accumulate leading to a higher impact of delay on the trial design.

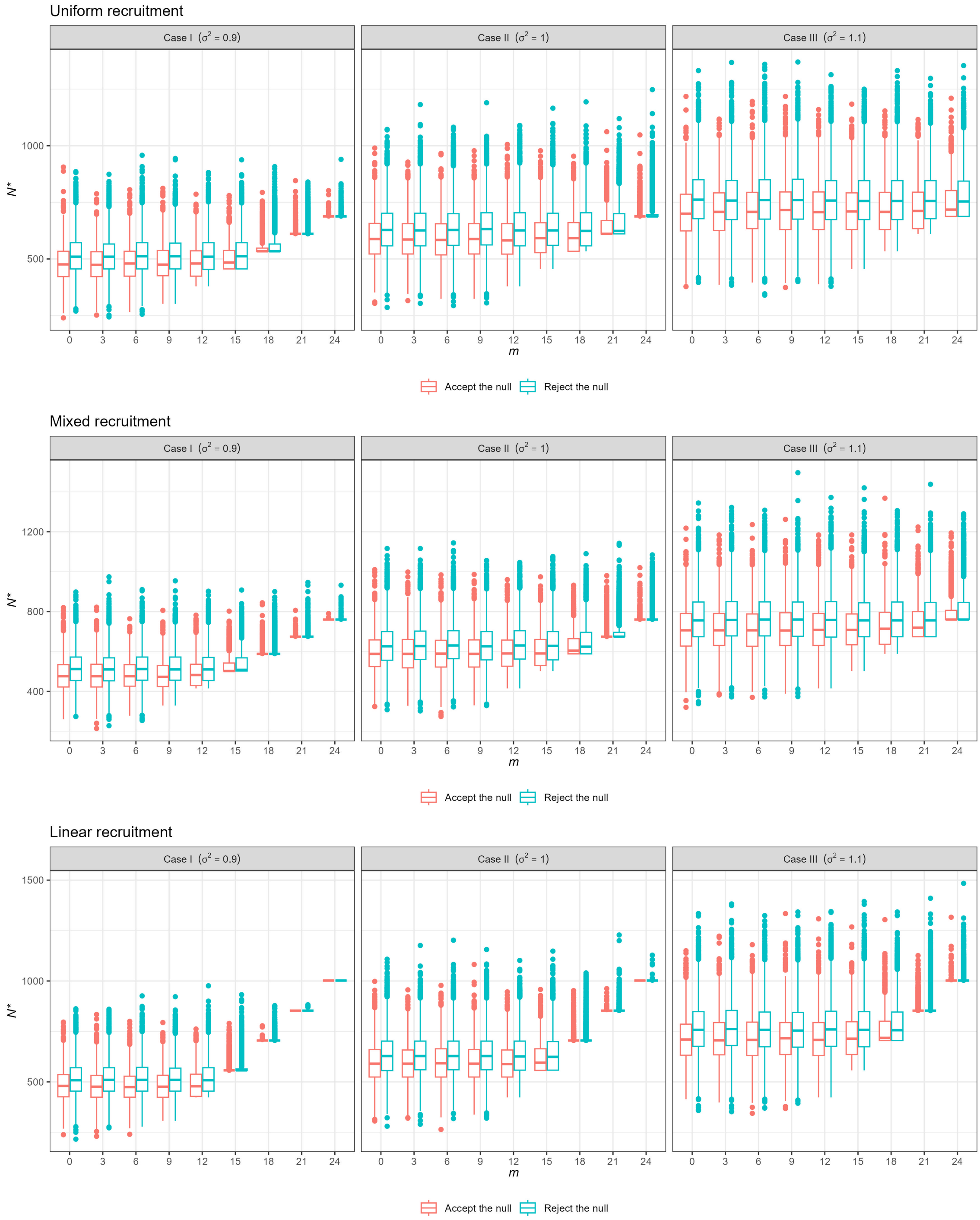

Figure 1: Distribution of the final sample size based on the decision to reject or accept the null under varying delay lengths for uniform, mixed and linearly respectively (top to bottom) increasing recruited samples and different values of σ^2 for a treatment effect of $\delta = \tau = 0.2$. In this case, the initially planned sample size is 618 across both arms, the oracle sample sizes are 500, 618 and 748 respectively for cases I, II and III.

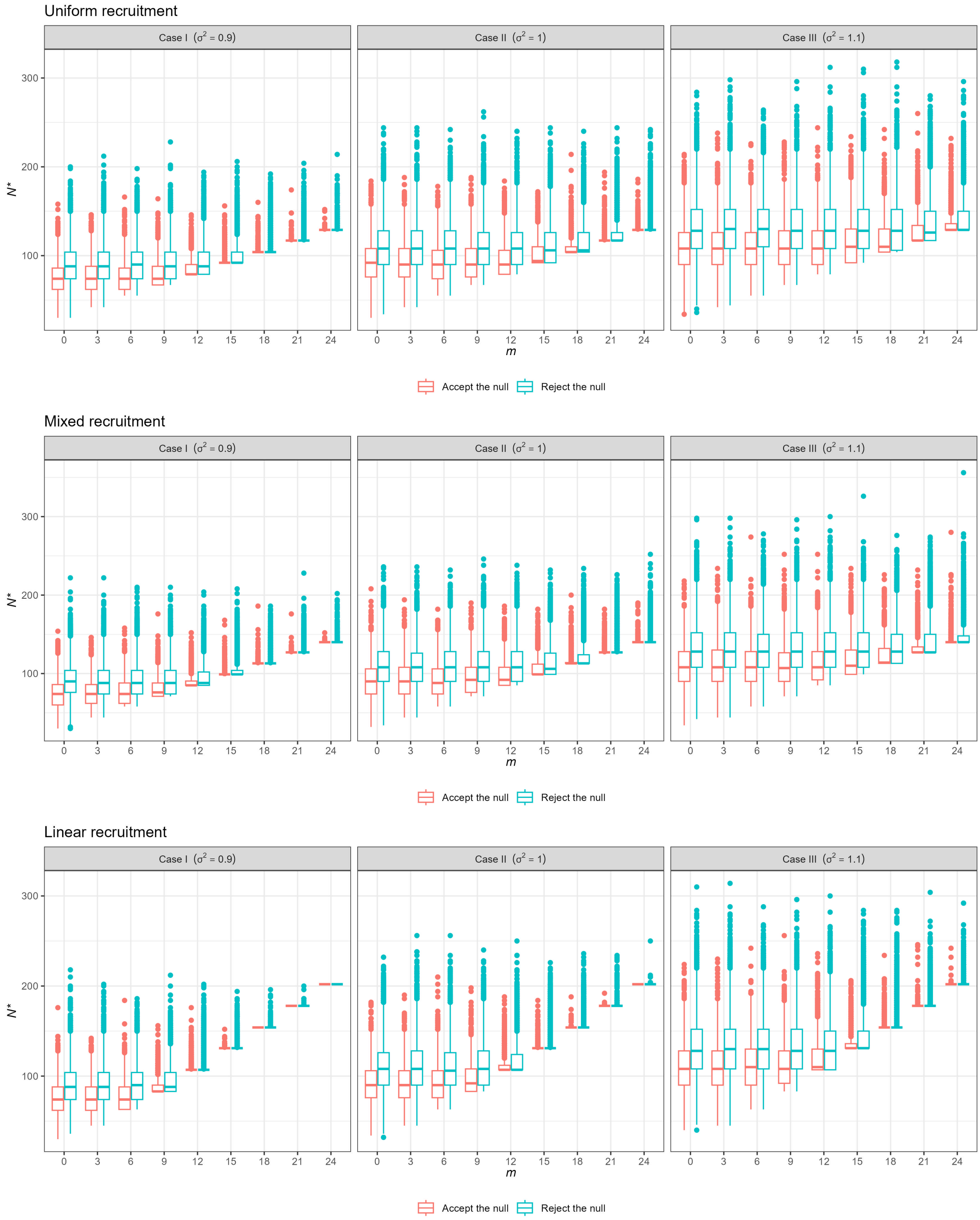

Figure 2: Distribution of the final sample size based on the decision to reject or accept the null under varying delay lengths for uniform, mixed and linearly respectively (top to bottom) increasing recruited samples and different values of σ^2 for a treatment effect of $\delta = \tau = 0.5$. Here, the first stage sample size is 15 in each arm, the initially planned sample size is 98. The oracle sample sizes are 80, 98 and 120 for cases I, II and III respectively.

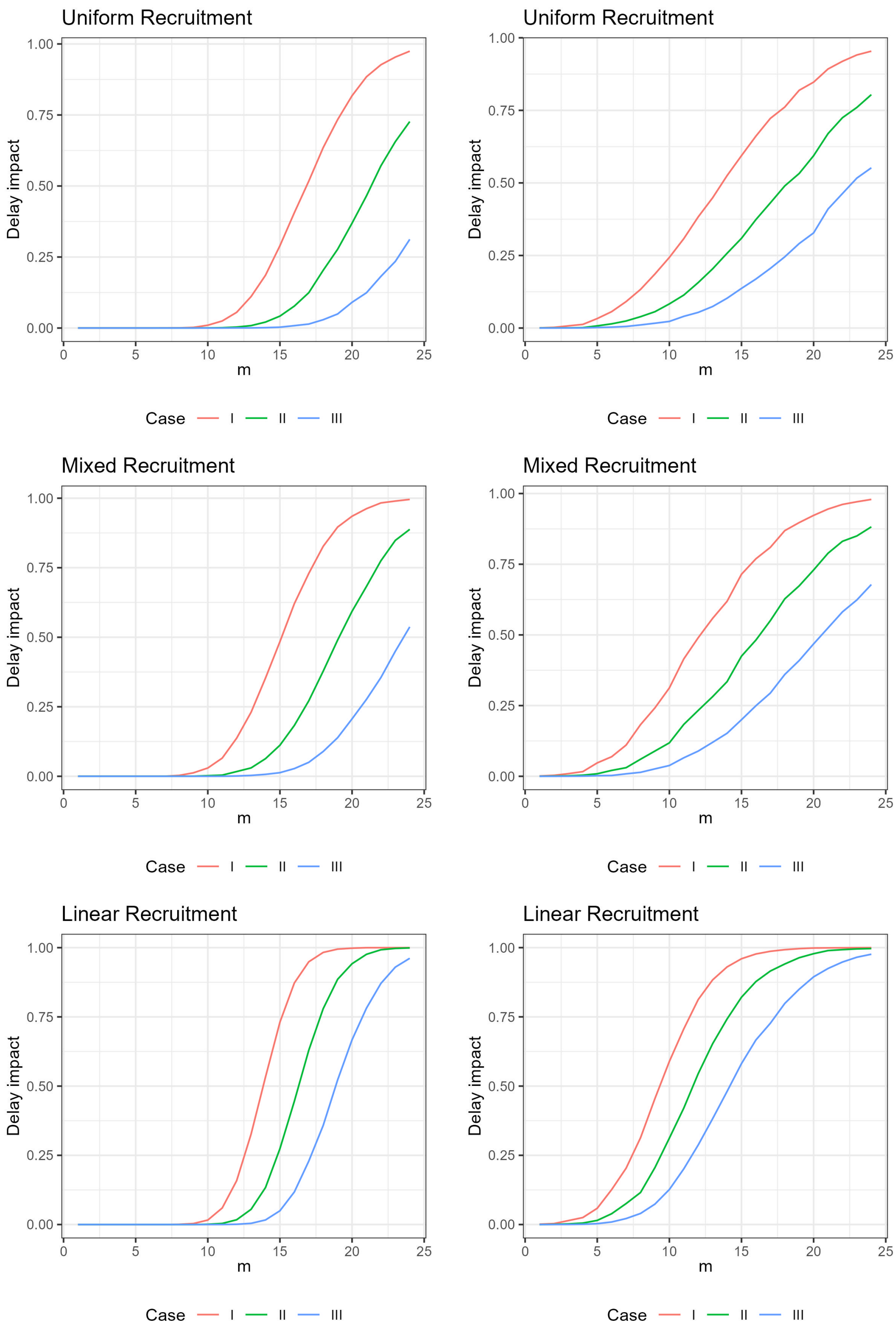

(a) $\delta = 0.2$

(b) $\delta = 0.5$

Figure 3: Delay impacts for varying delay lengths for different treatment effects of 0.2 and 0.5 respectively.

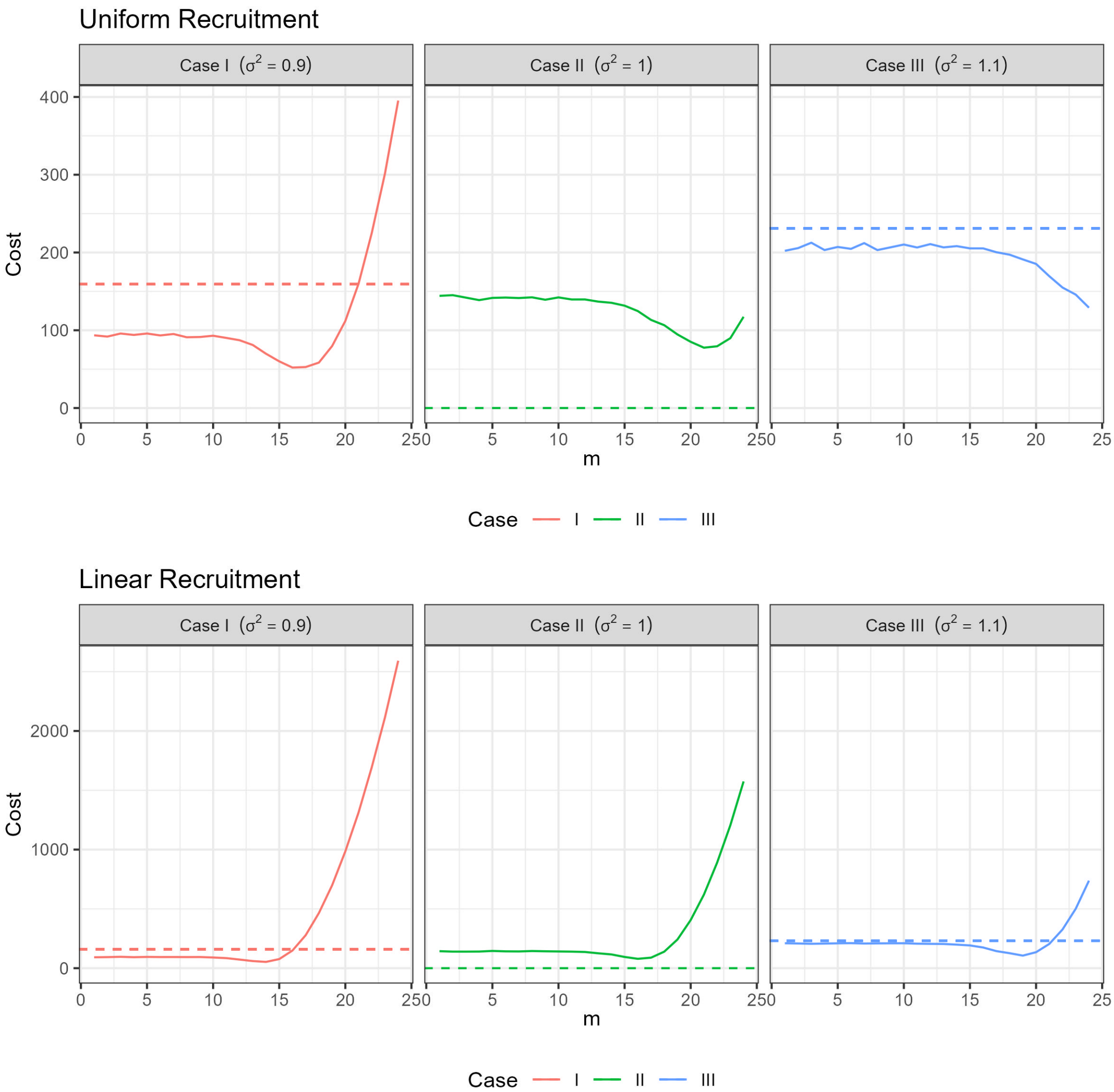

Figure 4: The 'Cost' for varying delay lengths ($m = 1, 2, \ldots, 24$), for $\sigma^2 = 0.9, 1, 1.1$, under uniform and linear recruitment patterns for $\delta = \delta_0 = 0.2$.

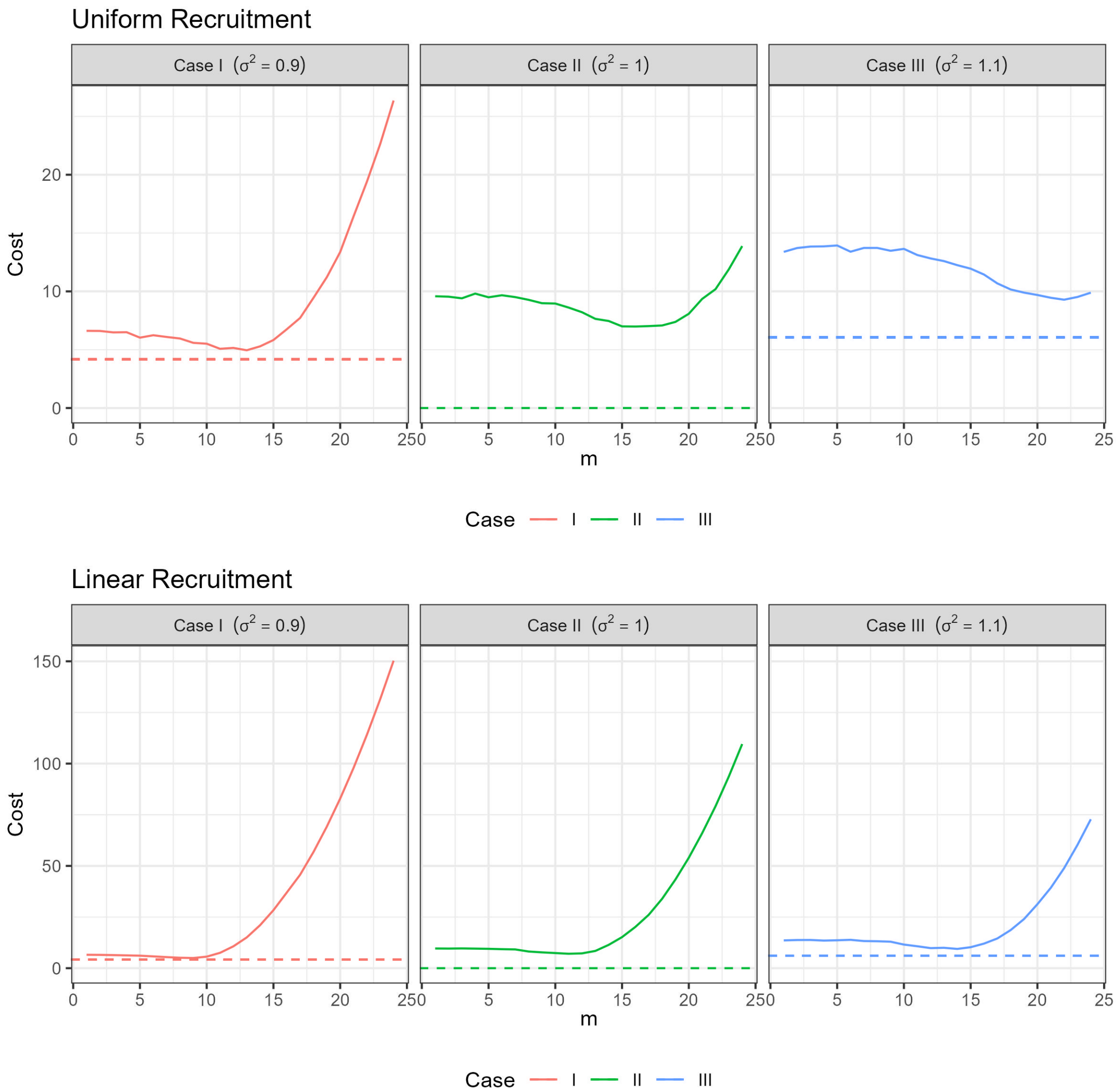

Figure 5: The ‘Cost’ for varying delay lengths ($m = 1, 2, \ldots, 24$), for $\sigma^2 = 0.9, 1, 1.1$, under uniform and linear recruitment patterns for $\delta = \delta_0 = 0.5$.

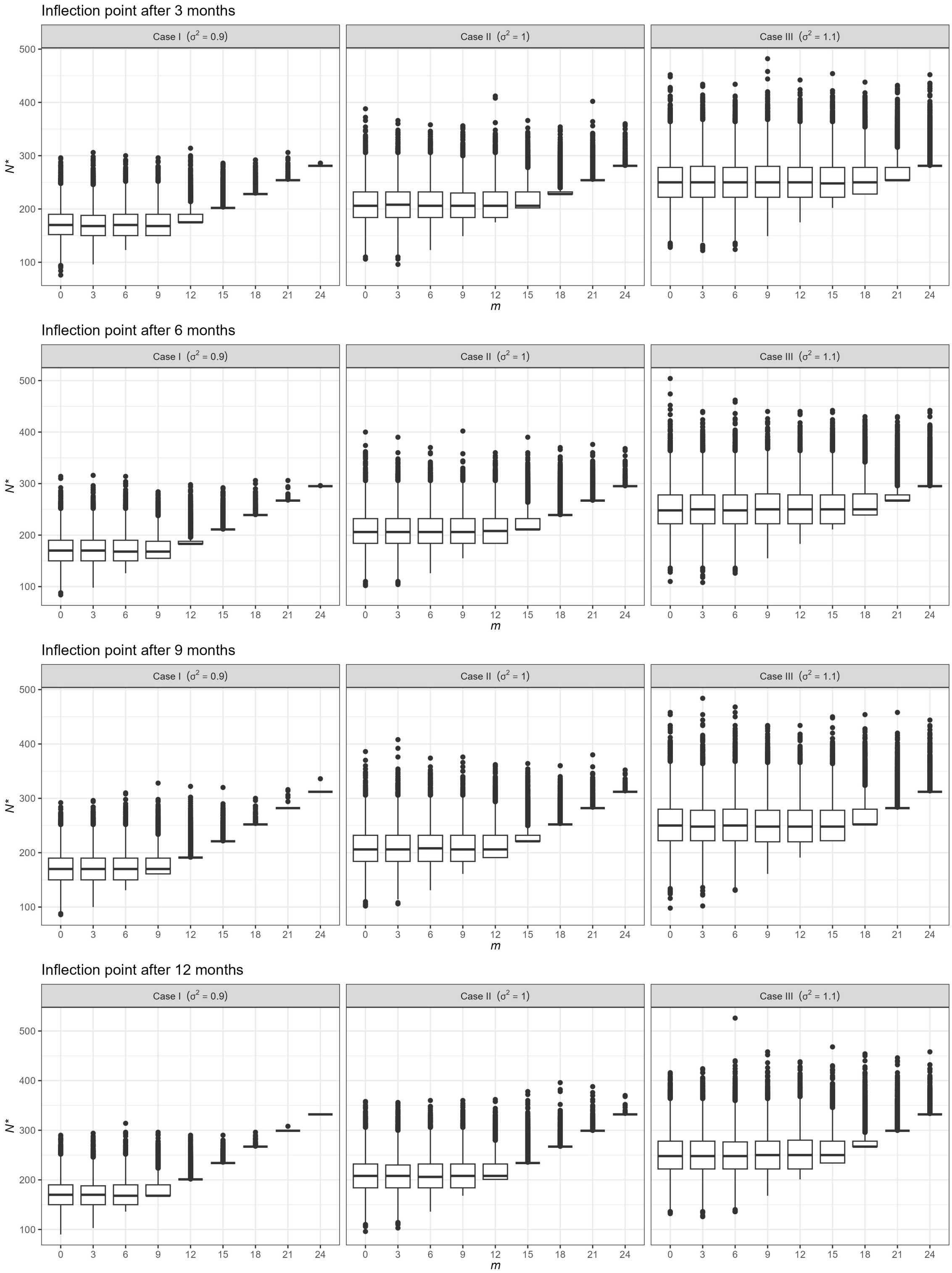

Figure 6: Distribution of the final sample size under varying delay lengths for a mixed recruitment pattern for different inflection points (the time points from which linear recruitment becomes uniform). Here, the treatment effect is assumed to be $\delta = \delta_0 = 0.35$ with σ^2 being 0.9, 1 and 1.1 respectively for cases I,II and III. Here, the first stage sample size is 35 in each arm, the initially planned sample size is 202.

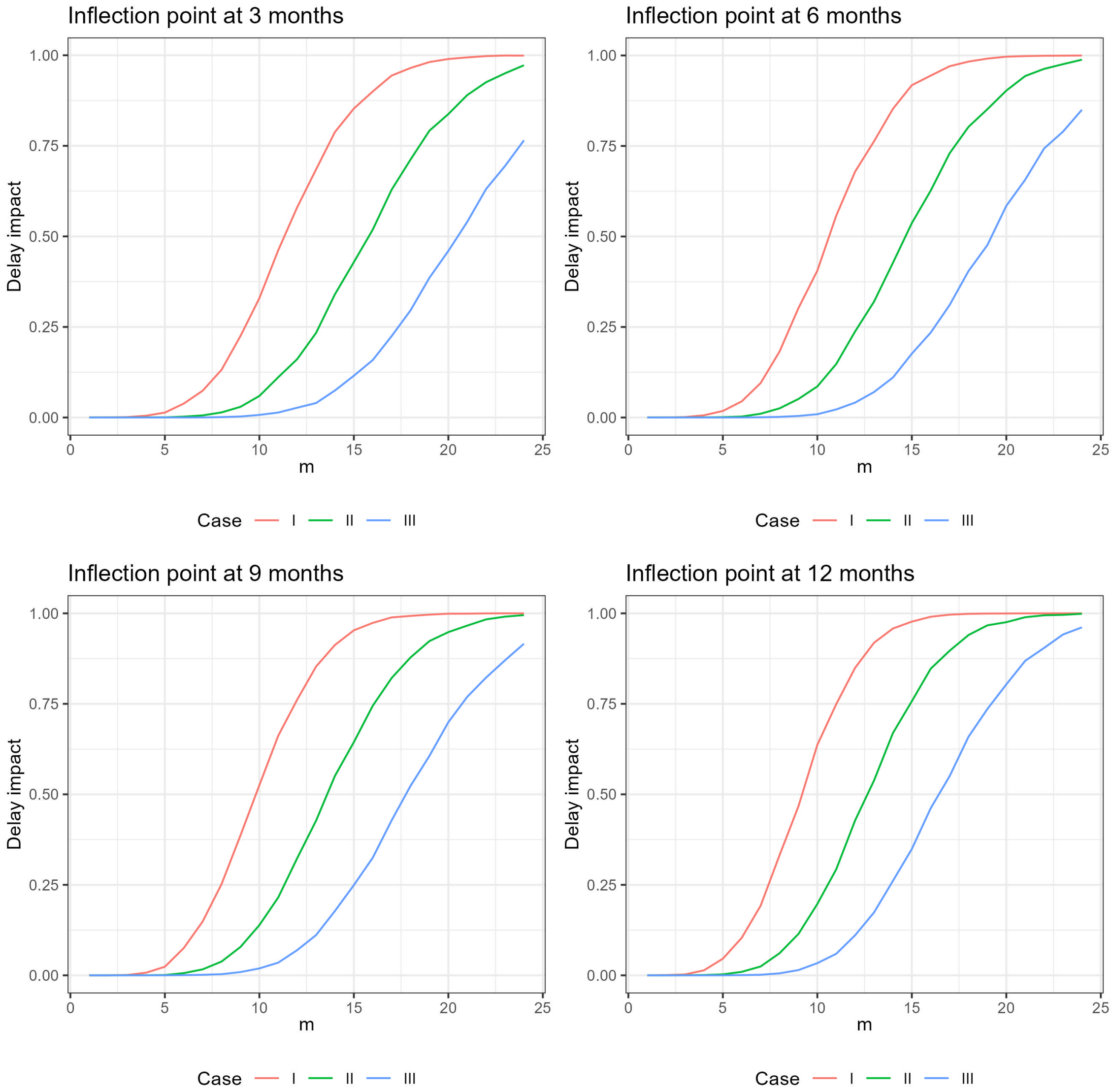

Figure 7: The '*delay impact*' for varying delay lengths ($m = 1, 2, \ldots, 24$) for $\sigma^2 = 0.9, 1, 1.1$, under mixed recruitment pattern for different inflection points.

4 Impact of delay on SSR design with binary treatment outcomes

4.1 SSR for binary treatment outcome

The paper describes in detail SSR for continuous outcome variables. For a binary response variable, the required sample size depends not only on the specified values of the type I error rate, power, and clinically relevant difference and the nuisance parameter, but also on the precise underlying success probabilities in the two arms. Friede and Kieser [2] proposed a blinded SSR process to re-estimate the sample size in this scenario. We present the effect of delay on such designs in this section.

Specifically, let us consider a clinical trial comparing two treatments based on a binary outcome variable. The success rates in each group are denoted as π_1 and π_2 respectively. Suppose there are to be $n/2$ samples observed in each treatment arm such that the total sample size for the trial is n. X_i, the number of successes in group $i = 1, 2$, is then binomially distributed with parameters $n/2$ and π_i. The parameter of interest is the absolute difference in the success probabilities, $\delta = \pi_2 - \pi_1$, and the hypotheses under test at level α and power $(1-\beta)$ for $\delta = \delta_1$ are assumed to be $H_0 : \delta \leq 0$ versus $H_0 : \delta > 0$. A normal approximation test can be used to test the above hypotheses, with the test statistic

$$U = \sqrt{\frac{1}{2} * \frac{n}{2}} \frac{\hat{\pi}_2 - \hat{\pi}_1}{\sqrt{\overline{\pi}(1-\overline{\pi})}},$$

where $\hat{\pi}_i = \frac{X_i}{n/2}$ are the observed proportions of successes in the arms and $\overline{\pi}$ is the observed pooled success rate across arms, computed as $\frac{X_1+X_2}{n}$. We reject the null at significance level α if $U > \Phi^{-1}(1-\alpha)$ [1].

The sample size required for the above test for a significance level of α and power of $1-\beta$ is typically computed as

$$n = 2 * \frac{[\Phi^{-1}(1-\alpha)\sqrt{2\bar{p}(1-\bar{p})} + \Phi^{-1}(1-\beta)\sqrt{p_1(1-p_1) + p_2(1-p_2)}]^2}{(p_2 - p_1)^2}, \tag{1}$$

where p_1 and p_2 are pre-specified estimates of π_1 and π_2 and $\bar{p} = (p_1 + p_2)/2$ [1].

As before, if the true values of π_1 and π_2 were known then the oracle sample size derived from the above formula, n_{oracle}, can be readily calculated. However, at the planning stage the values of the π_i are unknown and the p_i used in the calculation may be subject to substantial uncertainty.

Let us assume that the initial sample size estimate for the trial is n_{init} based on equation 1. After n_1 patients have been recruited in both the control and the treatment arm, we estimate the value of the pooled success rate and re-estimate the sample size based on this. Usually, n_1 is considered to be a fraction of n_{init} or pre-specified at the design stage. By estimating only a pooled success rate the blinding of the treatment allocations can remain intact. Here, the pooled success rate can be estimated as $p = (X_{11} + X_{12})/n_1$, where, X_{11} and X_{12} denotes the total number of successes in

the first stage, and the re-estimated sample size ($n_* = n_1 + n_{2*}$) is given as

$$n_* = 2 * \frac{\{\Phi^{-1}(1-\alpha) + \Phi^{-1}(1-\beta)\}^2}{\delta_1^2} 2p(1-p). \tag{2}$$

Note that, the individual values of X_{11} and X_{12} is often not required and the SSR can be performed from knowing the sum, $(X_{11} + X_{12})$ instead, retaining the blinding and integrity of the trial.

In the presence of delay, though, the re-estimated sample size is impacted by the pipeline observations, as described previously in the main article in Section "Sample size re-estimation". The final sample size (N^*) for the trial can be obtained from Equation (2) of the main article. The pipeline subjects (n_{delay}) are computed similar to Section "Computing the number of pipeline participants" , where, the total recruitment time is assumed to be t units (equal to 24 months in our simulations) to recruit all of n_{init} patients determined in the planning stage.

4.2 Results: Impact of delay on sample size re-estimation for a binary outcome variable

For a binary outcome variable, we assumed that initially the sample size calculations are based on a control treatment success rate of $\pi_1 = p_1 = 0.3$ and to detect a treatment effect of $\delta_1 = 0.25$ (i.e., $\pi_2 = p_2 = 0.55$). For this scenario, the trial requires a total of $n_0 = 94$ patients across both arms for a one-sided 5% significance level and 80% power. The total recruitment time was assumed to be 24 months to recruit all 94 patients and patient accrual was based on uniform or linear recruitment.

Similar to the result section in the main article, three scenarios were investigated, where,

- Case I: $\pi_1 = 0.1$.
- Case II: $\pi_1 = 0.3$.
- Case III: $\pi_1 = 0.5$.

In each case, $\pi_2 = \pi_1 + \delta_1$.

We have considered varying $m = 0, 1, \ldots, 24$ months. For each parameter combination, 10,000 simulations were run to obtain the distribution of N^*. For each simulation, the first $n_1 = 30$ [approximately 33% of n_{init}] samples across both arms were drawn from $Bin(1, \pi_1)$ and $Bin(1, \pi_2)$ populations, and the pooled sample success rate (p) was computed, based on which the sample size was re-estimated. The final sample size, N^*, was obtained through Equation (2) in the main article. For the simulation purposes we have not imposed a maximum allowed sample size in order to observe the full distribution of N^*.

4.2.1 Approach 1: Impact of delay on the distribution of re-estimated sample size

First we plot the distribution of the final sample size post SSR for varying delay lengths, $m = 0, 3, 6, \cdots, 24$. Figure 8 and Figure 9 shows N^* obtained through SSR for different m's. As observed

for a continuous outcome, the spread of the distribution of N^* reduces with increasing m. This is due to the distribution being truncated at $N^* = n_1 + n_{\text{delay}}$.

Similar to the continuous case, the most severe impact of delay is observed for case I, when $\pi_1 = 0.1$. Here, the required oracle sample size ($n_{oracle} = 66$) is less than the initially estimated required sample size ($n_{init} = 94$). Therefore, the delay period often results in accruing more patients than are estimated as being required and the greater the delay is, the more pipeline patients will be contributing to the loss of efficiency. Linearly increasing recruitment worsens the situation due to a higher number of pipeline patients.

The *delay impact* is also plotted in Figure 10 for the above simulation scenario. It is interesting to note that the impact of delay on the considered trials with a binary outcome is more severe compared to the continuous outcome examples from earlier. A reason for this may be the relatively smaller sample sizes required for the trials considered here compared to those in the normal outcome case.

Furthermore, it can be seen from Figure 9 that here N^* takes a single value of 104 (here, $n_1 = 30$ and $n_{delay} = 74$) for $m = 18$. Here, due to the recruited pipeline patients the final sample size already surpasses the the maximum sample size possible (100) for any $\pi \in (0, 1)$ for $\alpha = 0.05$ and $\beta = 0.2$ (due to the binary nature of the data). Thus for all simulation, the final sample size takes the constant value of 104, making *delay impact= 1.* Now if m increases further, this value would increase rapidly with a higher value of N^* consisting of $N^* = n_1 + n_{\text{delay}}$. Thus we see for linear recruitment the *delay impact* also quickly converges to 1 in Figure 10, where, for $m > 15$ the final sample size only takes the value $N^* = n_1 + n_{\text{delay}}$ with *delay impact*= 1.

4.2.2 Approach 3: Impact of delay on the cost metric

Here, we plot the cost metric to understand the impact of delay on blinded SSR for a binary outcome variable. Since similar inferences can be drawn from the cost metric plot alone, in this case, we have omitted the RMSE plot.

It is evident from Figure 11 that an increase in delay length significantly impacts the cost of the trial. It is also sensitive to the true value of the π_i. The trials where $\pi_1 = 0.1$ suffer higher costs in the presence of large delay ($m > 15$ months) due to a smaller oracle sample size, aligning with the previous inferences in the normal outcome case. Furthermore, also similar to prior observations, a linearly increasing recruitment rate increases the cost in comparison to uniformly recruited patients, as this results in a greater number of pipeline subjects.

The exact values for the *delay impact*, cost, and MSE of the SSR designs with binary outcomes can be found in the following Tables 4 and 5.

The following section contains the exact values of for *delay impact*, MSE, the cost metric and empirical power values for different m values (in particular, $m = 0, 3, 6, \ldots, 24$). Table 4 provides these values along with the oracle sample size and average final sample sizes from the 10,000 simulation under a uniform recruitment assumption, whereas, table 5 provides the same for a linearly

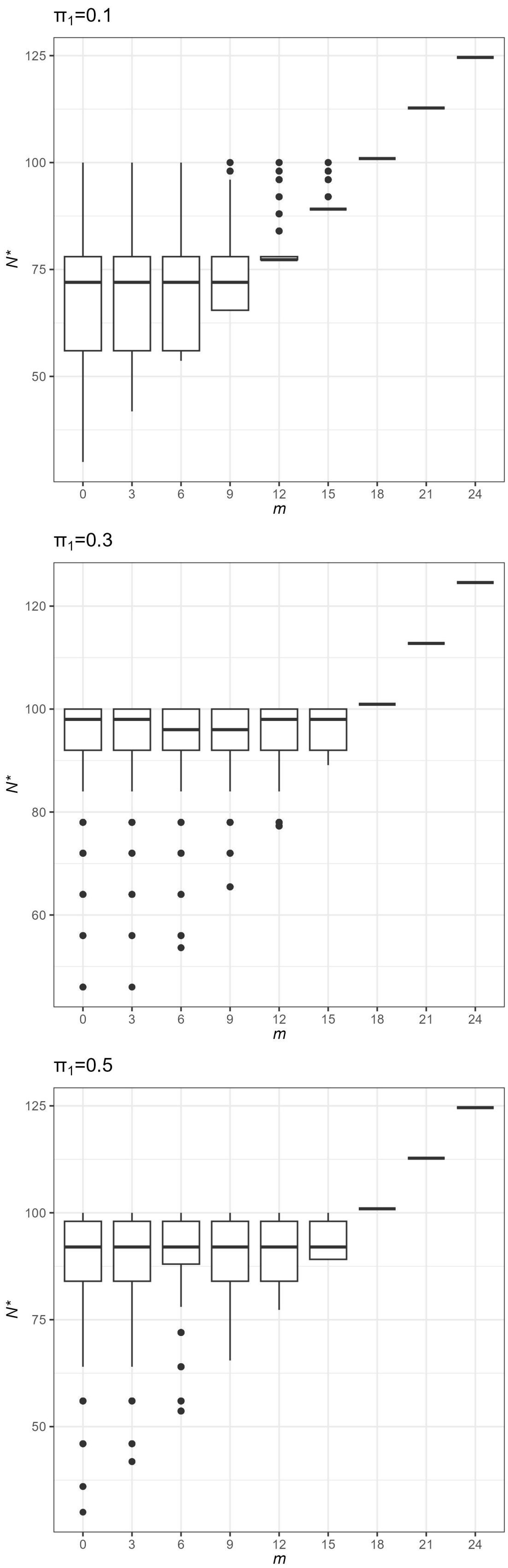

Figure 8: Distribution of the re-estimated sample size for varying delay lengths, under uniformly recruited samples, for different values of π_1.

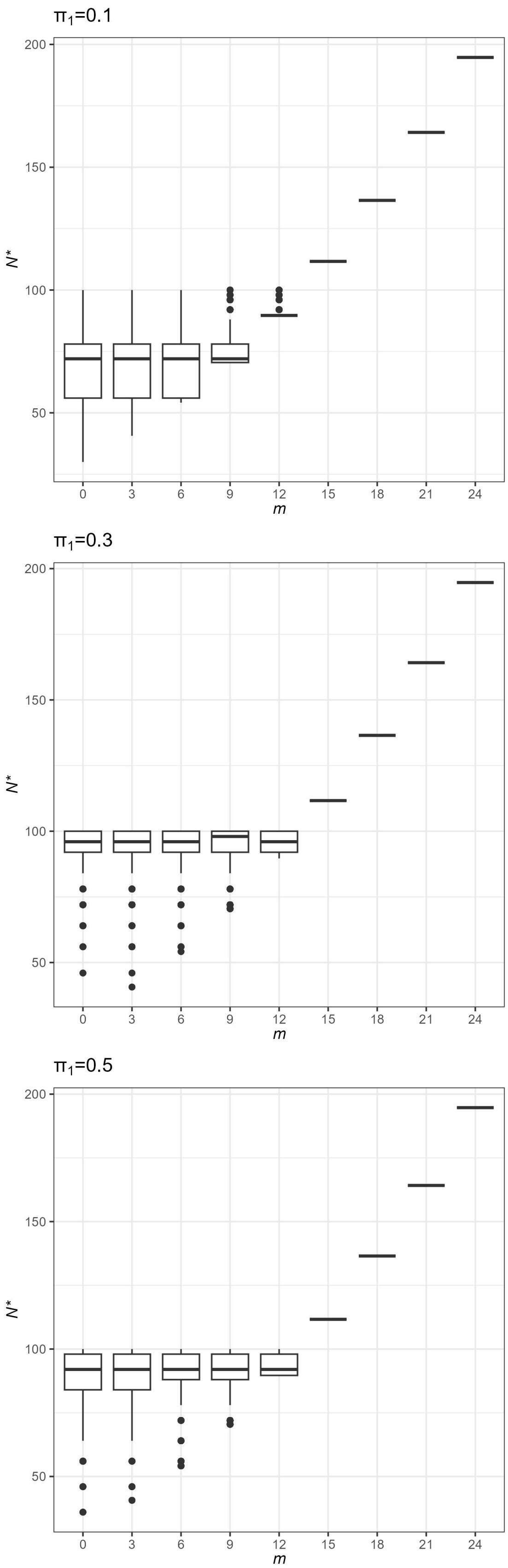

Figure 9: Distribution of the final sample size for varying delay lengths, under samples recruited at a linearly increasing rate, for different values of π_1.

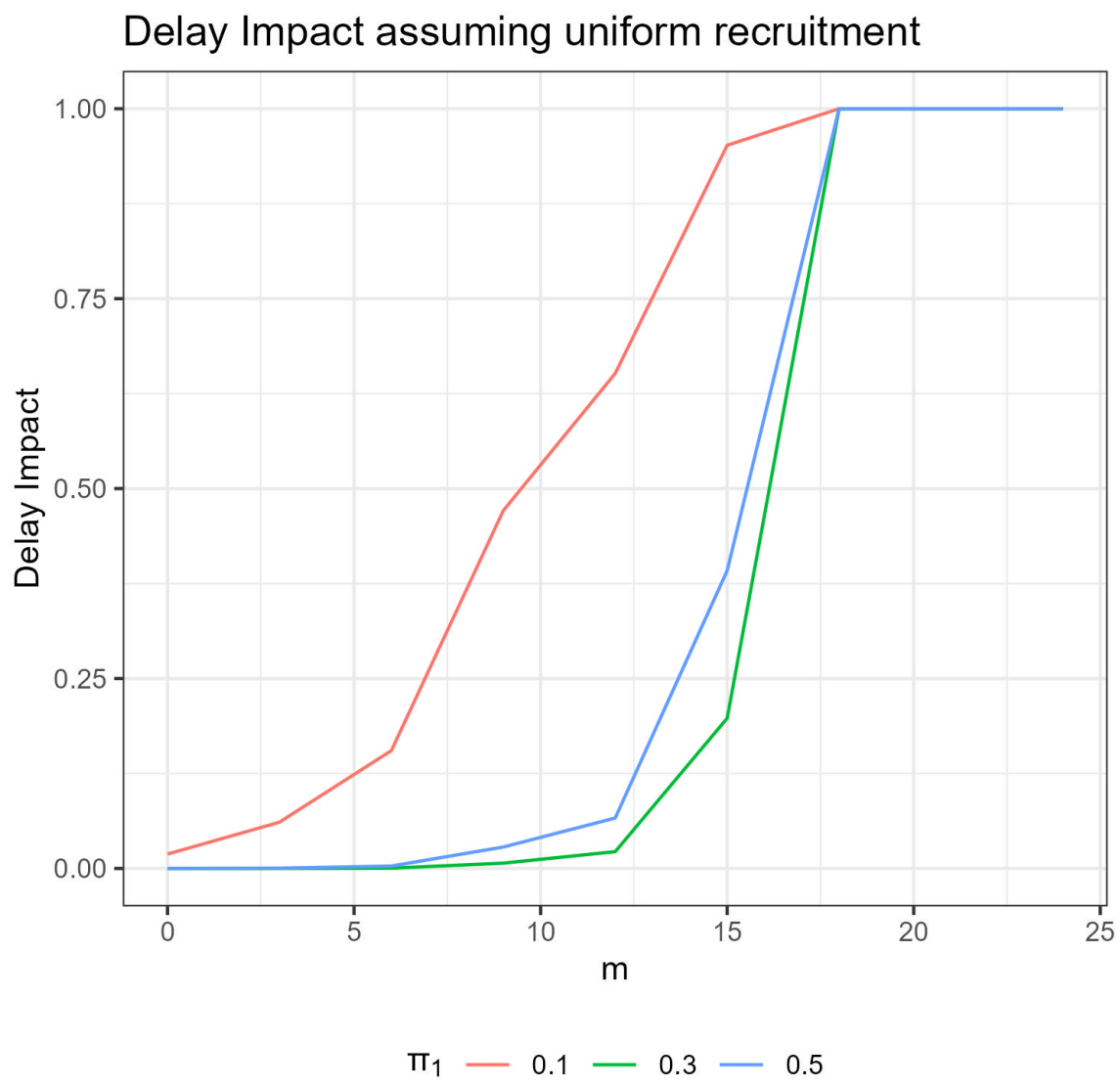

(a) Uniform recruitment.

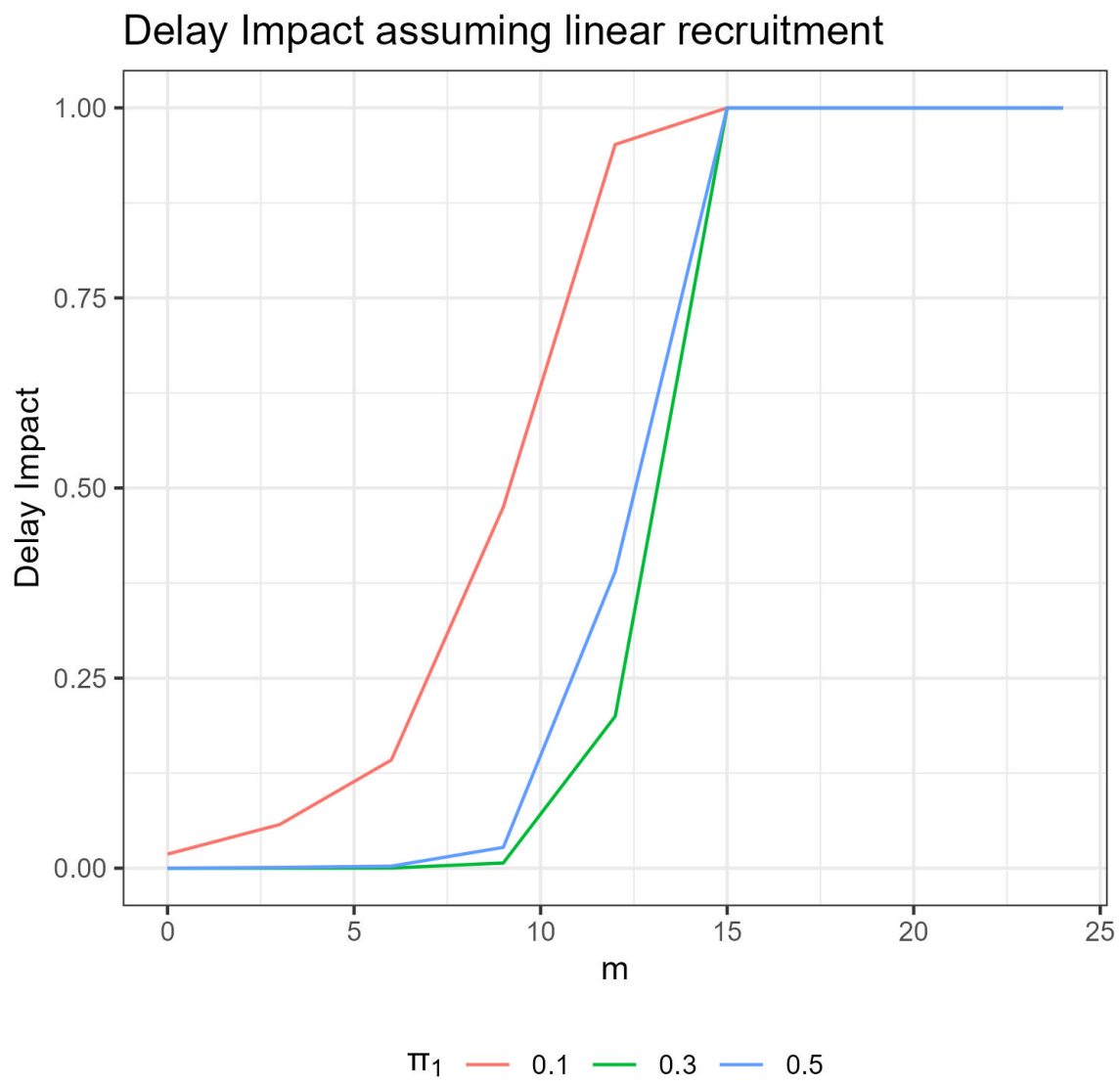

(b) Linear recruitment.

Figure 10: *Delay impact* for varying delay lengths ($m = 1, 2, \ldots, 24$) for $\pi_1 = 0.1, 0.3, 0.5$, under uniform and linear recruitment patterns.

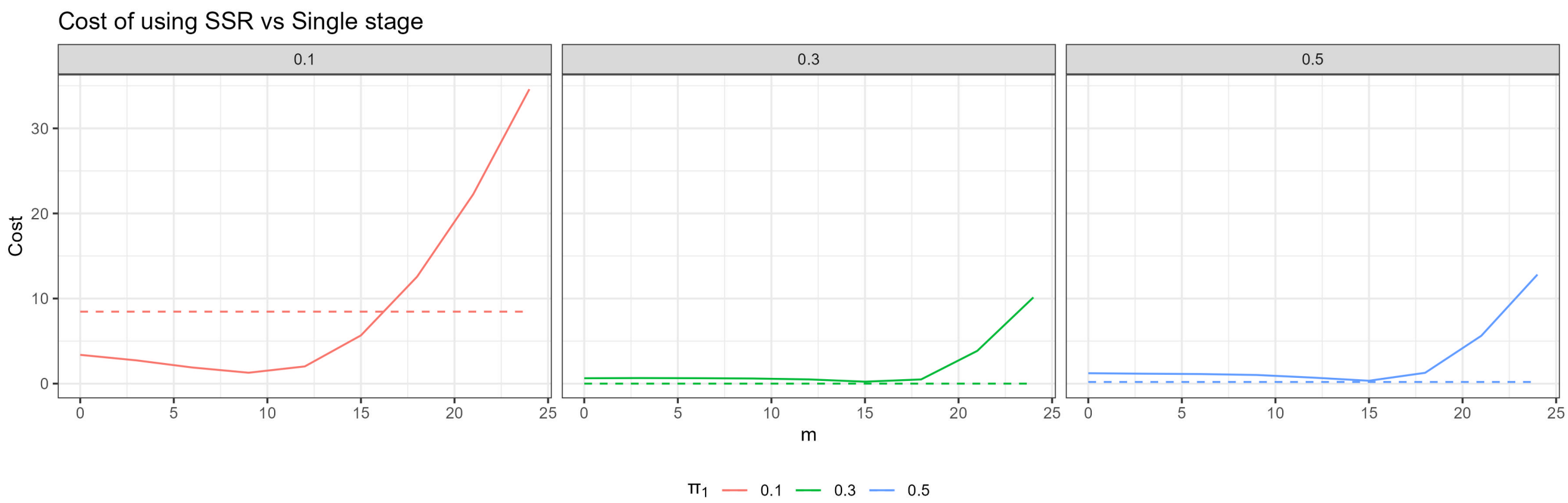

(a) Uniform recruitment.

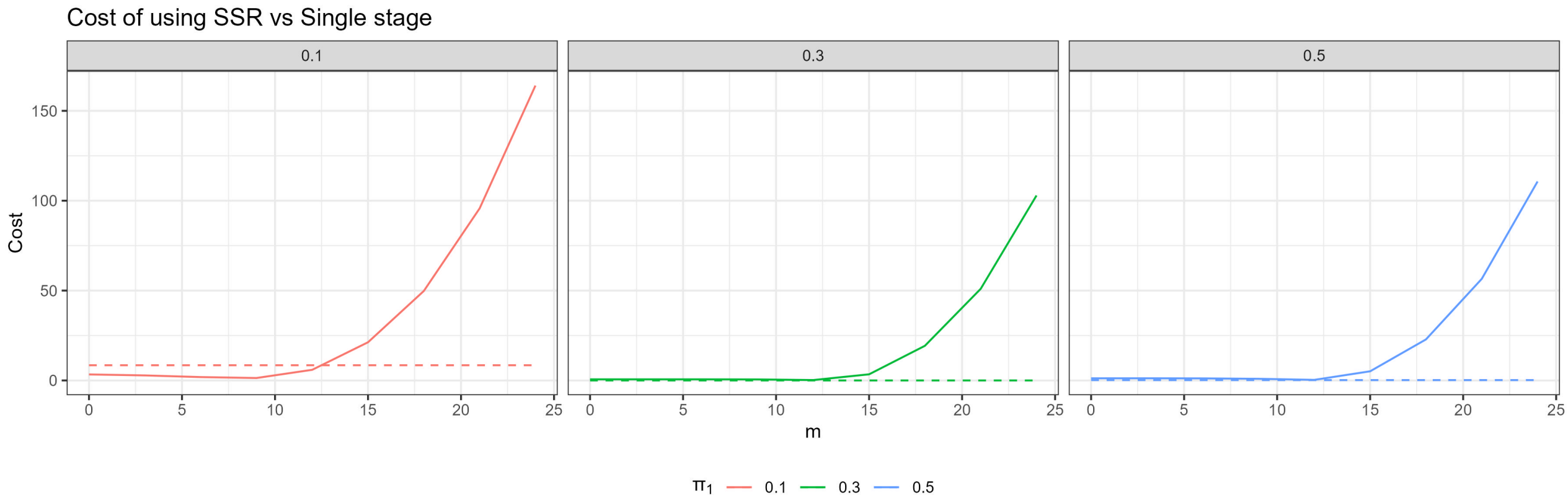

(b) Linear recruitment.

Figure 11: Cost for varying delay lengths for different values of $\pi_1 = 0.1$, 0.3 and 0.5 for uniform and linear recruitment patterns compared to a single stage design assuming $p_1 = 0.3$.

increasing recruitment pattern.

Table 4: Impact of delay on Efficiency and cost parameters for a SSR design with uniform recruitment for a binary outcome variable. Here, n_{single} is computed based on $\pi_1 = p_1 = 0.3, \delta_0 = 0.25$ and $\alpha = 0.05, \beta = 0.2$ and found to be 94 patients

π_1	Average p	Empirical Power	m	n_{oracle}	n_{delay}	Avg. N^*	MSE_{single}	MSE_{SSR}	$Cost_{single}$	$Cost_{SSR}$	Delay Impact
0.1	0.216	0.793	0	66.872	0.000	67.699	767.704	238.101	8.457	3.381	0.019
	0.217	0.796	3		11.822	68.246		208.562		2.734	0.061
	0.219	0.815	6		23.645	69.439		156.880		1.892	0.155
	0.219	0.834	9		35.467	72.943		112.741		1.280	0.471
	0.221	0.874	12		47.290	79.403		175.656		2.020	0.652
	0.222	0.904	15		59.112	89.341		506.203		5.668	0.952
	0.224	0.933	18		70.935	100.935		1,160.258		12.566	1.000
	0.223	0.955	21		82.757	112.757		2,105.435		22.256	1.000
	0.224	0.968	24		94.580	124.580		3,330.153		34.607	1.000
0.3	0.424	0.797	0	94.580	0.000	94.554	0.000	45.680	0.000	0.631	0.000
	0.424	0.799	3		11.822	94.460		47.407		0.654	0.000
	0.423	0.795	6		23.645	94.548		46.588		0.637	0.000
	0.423	0.793	9		35.467	94.462		44.753		0.602	0.007
	0.423	0.793	12		47.290	94.708		37.761		0.494	0.022
	0.423	0.800	15		59.112	95.702		18.386		0.229	0.198
	0.420	0.821	18		70.935	100.935		40.387		0.491	1.000
	0.422	0.861	21		82.757	112.757		330.423		3.847	1.000
	0.423	0.885	24		94.580	124.580		900.000		10.133	1.000
0.5	0.628	0.802	0	90.622	0.000	90.553	15.665	87.060	0.192	1.223	0.000
	0.628	0.798	3		11.822	90.597		83.520		1.165	0.000
	0.627	0.798	6		23.645	90.697		81.926		1.124	0.003
	0.627	0.795	9		35.467	90.775		76.166		1.015	0.028
	0.626	0.808	12		47.290	91.324		55.382		0.705	0.066
	0.622	0.811	15		59.112	93.646		27.930		0.337	0.392
	0.619	0.847	18		70.935	100.935		106.357		1.271	1.000
	0.621	0.867	21		82.757	112.757		489.975		5.620	1.000
	0.623	0.901	24		94.580	124.580		1,153.135		12.815	1.000

Table 5: Impact of delay on Efficiency and cost parameters for a SSR design with linear recruitment for a binary outcome variable. Here, n_{single} is computed based on $\pi_1 = p_1 = 0.3, \delta_0 = 0.25$ and $\alpha = 0.05, \beta = 0.2$ and found to be 94 patients

π_1	Average p	Empirical Power	m	n_{oracle}	n_{delay}	Avg. N^*	MSE_{single}	MSE_{SSR}	$Cost_{single}$	$Cost_{SSR}$	Delay Impact
0.1	0.215	0.783	0	66.872	0.000	67.631	767.704	235.505	8.457	3.343	0.019
	0.217	0.803	3		10.657	68.179		211.462		2.798	0.057
	0.220	0.819	6		24.151	69.915		156.259		1.870	0.142
	0.222	0.850	9		40.483	75.310		118.731		1.352	0.475
	0.222	0.904	12		59.652	89.861		529.678		5.921	0.952
	0.222	0.956	15		81.659	111.659		2005.827		21.243	1.000
	0.224	0.975	18		106.503	136.503		4848.386		49.783	1.000
	0.225	0.991	21		134.184	164.184		9469.555		95.733	1.000
	0.224	0.996	24		164.703	194.703		16340.590		164.059	1.000
0.3	0.424	0.798	0	94.580	0.000	94.548	0.000	45.228	0.000	0.615	0.000
	0.423	0.803	3		10.657	94.563		45.191		0.615	0.000
	0.423	0.791	6		24.151	94.572		45.137		0.615	0.000
	0.424	0.792	9		40.483	94.568		42.639		0.568	0.007
	0.423	0.800	12		59.652	95.860		17.582		0.218	0.200
	0.419	0.854	15		81.659	111.659		291.692		3.408	1.000
	0.424	0.911	18		106.503	136.503		1757.528		19.270	1.000
	0.424	0.947	21		134.184	164.184		4844.740		50.976	1.000
	0.424	0.972	24		164.703	194.703		10024.583		102.899	1.000
0.5	0.628	0.797	0	90.622	0.000	90.679	15.665	85.139	0.192	1.192	0.000
	0.629	0.796	3		10.657	90.558		86.006		1.205	0.001
	0.628	0.791	6		24.151	90.665		83.666		1.148	0.003
	0.628	0.800	9		40.483	90.884		70.184		0.919	0.027
	0.621	0.808	12		59.652	93.804		26.793		0.323	0.390
	0.615	0.858	15		81.659	111.659		442.548		5.093	1.000
	0.623	0.920	18		106.503	136.503		2105.041		22.827	1.000
	0.624	0.958	21		134.184	164.184		5411.369		56.518	1.000
	0.623	0.979	24		164.703	194.703		10832.790		110.691	1.000

References

[1] Meinhard Kieser, Methods and Applications of Sample Size Calculation and Recalculation in Clinical Trials, http://www.springer.com/series/15122, Springer Series in Pharmaceutical Statistics, 2020, https://doi.org/10.1007/978-3-030-49528-2

[2] Tim Friede and Meinhard Kieser, Sample size recalculation for binary data in internal pilot study designs, Pharmaceutical Statistics, 10.1002/pst.140, 269-279, 3, 2004
